# Universal mental health training for frontline professionals (UMHT)’s feasibility analysis

**DOI:** 10.12688/openreseurope.17358.3

**Published:** 2025-03-21

**Authors:** Viktoriia Gorbunova, Vitalii Klymchuk, Philip Santangelo

**Affiliations:** 1 Department of Behavioural and Cognitive Sciences, University of Luxembourg, Esch-sur-Alzette, Luxembourg, L-436, Luxembourg; 2 Department of Applied and Social Psychology, Ivan Franko Zhytomyr State University, Zhytomyr, Ukraine; 3 Department of Social Sciences, University of Luxembourg, Esch-sur-Alzette, Luxembourg, L-4365, Luxembourg

**Keywords:** mental health, universal mental health training, frontline professionals, mental disorders, mental crises

## Abstract

**Background:**

Universal Mental Health Training for Frontline Professionals (UMHT) is an educational programme developed and piloted in Ukraine in 2021-2023. The UMHT trains frontline professionals (FLPs) to interact with, support, and refer individuals with mental health conditions for professional help.

**Methods:**

To assess the UMHT feasibility in four focus areas (programme’s demand, acceptability, adaptability, and extendability), we used statistics on the actual use of the programme, as well as data from satisfaction and usability surveying of 144 programme deliverers and 714 trained frontline professionals. A combination of Kruskal-Wallis and Post Hoc Dunn tests was used to identify statistically significant intergroup differences in the UMHT usability.

**Results:**

Programme’s demand increased through years of implementation (2021, 2022, 2023) in terms of the numbers of training events (27, 35, 90), trained frontline professionals (596, 779, 1548), involved donors and supporters (1, 4, 9) and implementers (2, 10, 18). The UMHT acceptability as satisfaction with the programme content and delivery, measured out of 5, is 4.81 (SD=0.291) for the UMHT trainers and 4.78(SD=0.434) for trained FLPs. The UMHT preparedness to use trained skills after participation in the training events, on the same scale, is 4.57 (SD=0.438) for the UMHT trainers and 4.46 (SD=0.650) for trained FLPs. The highest levels of usability of all UMHT skills on a scale from 0 to 1 were found for educators (0.68 [SD=0.118]), police officers (0.67 [SD=0.098]), and social workers (0.66 [SD=0.113]).

**Conclusions:**

The UMHT offers a universal frame of interaction with people with mental health conditions for frontline professionals. Assessment of the UMHT feasibility shows the programme’s potential for further development and implementation. Programme trainers as its deliverers and frontline professionals as its recipients report high satisfaction with training content and delivery as well as preparedness to apply gained knowledge and skills in practice.

## Plain language summary

Universal Mental Health Training for Frontline Professionals (UNMHT) is an educational programme developed and piloted in Ukraine in 2021-2023 to bridge the mental health gap between Ukrainians’ needs in support and the answers from the health system. The UMHT trains frontline professionals to interact with, support, and refer individuals with mental health conditions for professional help. The UMHT offers a universal frame of interaction with people with mental health conditions for frontline professionals. Assessment of the UMHT feasibility shows the programme’s potential for further development and implementation. Programme trainers as its deliverers and frontline professionals as its recipients report high satisfaction with training content and delivery as well as preparedness to apply gained knowledge and skills in practice.

## Introduction

The Universal Mental Health Training for Frontline Professionals (UMHT) was developed in Ukraine ^1^ as an educational programme for police officers, emergency responders, social workers, educators, pharmacists, priests, and other professionals on the first line of service to the general public (see the detailed UMHT description and the rationale for implementing in Gorbunova *et al.*, 2024). Whereas their professional roles often imply working with people in difficult or unstable life circumstances, a high level of mental health awareness and skills to manage mental health issues are essential. The UMHT aims to equip frontline professionals with knowledge and skills to deliver evidence-based responses to the mental health needs of the population they serve using the UMHT five-step model: recognise, validate, support, refer, and ensure.

The feasibility of such public health programmes as the UMHT is one of the primary requirements for their implementation ( Bowen *et al.*, 2009). In every case, a feasibility study answers whether an intervention is appropriate for further development, testing and implementation and identifies if anything needs modification and adjustments. According to the Framework for the Development and Evaluation of Complex Interventions issued by the National Institute for Health and Care Research (NIHR), research-driving policymaking implies feasibility and pilot trials as steps taken immediately after intervention development prior to effectiveness evaluation ( Skivington *et al.*, 2021).

Among examples of widespread public mental health programmes for which the feasibility analysis was done in different settings are Psychological First Aid / PFA ( Brown *et al.*, 2009; Chandler *et al.*, 2023; Geoffrion *et al.*, 2023) Mental Health First Aid / MHFA ( Crooks *et al.*, 2018; Narayanasamy *et al.*, 2018) and Mental Health Gap Action Programme / mhGAP ( Mutiso *et al.*, 2019a; Mutiso *et al.*, 2019b; Siddaiah *et al.*, 2022). The main idea of the mentioned studies was to predict the future success of an intervention or to assess the current situation with its implementation in different populational or professional groups, in different countries and under different circumstances. Some studies focused on the feasibility of programme modifications, such as its transition into e-format or cultural adaptation. Measurements of feasibility analyses usually fall into two broad categories: 1) the feasibility of an intervention itself as its ability to be successful in terms of technical and content features and 2) the feasibility of an intervention’s implementation and maintenance in the real world with its risks and unpredictabilities ( Pfledderer *et al.*, 2024).

Keeping in mind the fact that public MH intervention is not just a scientific product but a scientific product for wide public usage, often linked to policymaking, it needs to be feasible in terms of design and technical features, operational strategy, and finance model, together with established risk management measures. Such qualities let a programme be considered in the public health domain as an instrument able to bridge the gap between a population’s needs and the society's responses.

The present study aims to assess the feasibility of the UMHT as a public mental health promotion and prevention intervention based on four focus areas (demand, acceptability, adaptability and extendability) derived from the Key areas of focus for feasibility studies and possible outcomes ( Bowen *et al.*, 2009). Feasibility is determined based on various aspects such as UMHT’s demand as well as users’ satisfaction and programme potential to be adjustable for different groups of frontline professionals and to be open to modifications due to design and content features.

### Research questions

1. **Demand**: to what extent is the UMHT likely to be used?
2. **Acceptability**: to what extent is the UMHT suitable, satisfying and attractive to programme deliverers and recipients?
3. **Adaptability**: to what extent is the UMHT suitable for adjusting content and procedure to new formats and working with different population groups?
4. **Extendability**: to what extent can the UMHT be expanded to cover new topics and solve new problems?

## Methods

### Study settings

UMHT delivery and data collection was made in Ukraine in collaboration with a team of academics from Zhytomyr State University with support of the “Mental health for Ukraine Project” (MH4U), implemented in Ukraine by GFA Consulting Group GmbH.

The UMHT was disseminated by the Training of Trainers (ToT) approach, highlighted in the mhGAP Operation Guide ( WHO, 2018). The training of trainers lasts seven full days and includes an overview of all UMHT modules, along with detailed training delivery methodology. It aims to prepare trainers to lead training for frontline professionals. Each training for frontline professionals consists of compulsory modules (Introductory module and Final module) and selective modules, each dedicated to specific mental health conditions. Each module lasts 90 minutes and includes the standard set of slides, examples, role-play exercises and discussions. Usually, 2–4 modules relevant to the targeted audience of FLPs’ needs are selected for the training. After the training, all the participants have supervision support.

To assess the feasibility of the UMHT, we surveyed the programme's satisfaction and usability by all of its trainers (mental health professionals trained to deliver the UMHT to different groups of frontline professionals) and recipients, *i.e.*, frontline professionals (FLPs). We analysed the answers of 144 UMHT mental health professionals intended to become UMHT trainers (100% response rate) and 714 out of 2726 frontline professionals (26,2% response rate). Among frontline professionals were social workers (n = 203), educators (n = 152), police officers (n = 122), employees of occupation centres (n = 58), emergency responders (n = 52), military volunteers (n = 34), pharmacists (n = 37), librarians and museum workers (n = 29), priests and clerics (n = 27). To find out which set of mental conditions should be included in the programme for every particular group of FLPs, we run preliminary interviews, asking their representatives about age, typical communication pathways, widespread problems and other features of people with whom they work. The distribution of mental health conditions (training modules) with numbers of trained FLPs whose answers were taken into the analysis is reflected in Table 1.

**Table 1.** Distribution of mental health conditions (training modules) and N of trainees in the trained groups of frontline professionals.

Groups of frontline professionals	educators	emergency responders	librarians and museum workers	military volunteers	pharmacists	police officers	priests and clerics	social workers	employees of occupation centres	Total
Mental health conditions (training modules)										
attention-deficit / hyperactivity disorder	75		14					28		117
autism spectrum disorder	78		15					36	29	158
delirium		24		14		44		27		109
depressive disorder	43		29		18		27	59	29	205
disruptive, impulse-control, and conduct disorders	35		14			44		52		145
elimination disorders	25									25
feeding and eating disorders	34									34
gambling disorder				20		30			29	79
illness anxiety disorder					37			71	29	137
intellectual disability	36		15			15	27	100	29	222
neurocognitive disorders					20			44		64
panic disorder	39	28		34	37	15	27	57		237
post-traumatic / acute stress disorder	18	52		34	34		54			192
separation anxiety disorder	50									50
sleep-wake disorders	17			28	37		27	23		132
social anxiety disorder	43							39	29	111
specific phobias and agoraphobia	17				36			34		87
substance-related disorders	53	28		68		14	54		58	275
aggressive behaviour						78				78
self-harm behaviour						80				80
suicide & life-threatening behaviour						63				63
unusual & disorganized behaviour						126				126
Total	563	132	87	198	219	509	216	570	232	2726

### Study design and data collection procedures

To answer the research questions, we carried out an analysis of various feasibility focus areas. The demand, acceptability, adaptability and extendability were assessed during the pilot roll-out in 2021-2023 in eight regions of Ukraine (Kyiv, Lviv, Rivne, Chernivtsi, Dnipro, Luhansk, Donetsk, and Zhytomyr oblasts), mainly through satisfaction and usability surveying of programme trainers and trainees (FLPs of different groups). A summary of the research methods and measurements is presented in Table 2.

**Table 2.** Research methods and measurements.

UMHT’s feasibility focus areas	Measurements	Research methods
**Demand**	Actual use of the programme	Usability statistic
**Acceptability**	Training content and delivery satisfaction for trainers and FLPs	Satisfaction surveying, accreditation structured assessment
**Adaptability**	Usability of the programme by different groups of FLPs	Usability surveying, supervision reports evaluation, case comparison
**Extendability**	Usability of the new modules (mental health crisis)	Usability surveying, supervision reports evaluation, case comparison

To assess the programme ***demand***, we measured the actual use of the programme with statistics on the number of trainings conducted with donor support in terms of organisation and financing and independently on request of different organisations.

As the programme ***acceptability*** measurement, we chose training content and delivery satisfaction by its trainers as deliverers and FLPs as its recipients through satisfaction surveying. All audiences were asked to assess on the 5-grade scales (ranging from 1-“no, not at all” to 5-“yes, strongly agree”) whether training materials were structured and transparent, whether the balance between theory and practice was kept, whether there were enough examples and explanations, whether answers to participants’ questions were full and clear; whether trainers were careful of sensitive topics and mindful of stigma and whether presentations and following material were of high quality and perceptibility.

The same rating scale was applied for self-assessing the ToT participants' post-training boost of preparedness to lead UMHT in terms of knowledge, skills and general readiness: *"My understanding of the topic of interactions with people with MH issues has increased"; "My skills to lead UMH training and supervisions was mastered"; "My readiness to lead UMH training and supervisions has increased"*. FLPs assessed their subjective preparedness to interact with people with MH conditions with the questions: *"My understanding of the topic of interactions with people with MH issues has increased"; "My skills to interact with people with MH issues was mastered; "My readiness to work with people with MH issues has increased".*

Also, future trainers and FLPs were asked to name in the free text entry knowledge and skills they would like to strengthen and suggest changes in the training materials and process to improve it. Additionally, data from the accreditation assessment of UMHT trainers' knowledge and skills observed in the process of training delivery was taken into account. In particular, we assessed subject knowledge, organisational skills, instrumental skills, motivational skills, and ethical skills.

The ***adaptability*** of the programme was measured through its comparative usability among different groups of FLPs, specifically with questions: *“Did you work with people with mental conditions after the UMHT?”; “Did you use the knowledge and skills gained during the UMHT?”; “If yes, what knowledge and skills gained during the UMHT you were able to use?”* The list of knowledge and skills consists of those needed on each step of the UMHT model and purposely targeted during the training: recognise MH conditions, validate MH conditions with a person / their caregivers, support a person, refer for professional help, and ensure the reference was successful.

Additional information, such as the content of supervision requests from the UMHT trainers, was derived from supervision reports. In general, there were six possible types of supervision: organisational supervision (issues related to the organization of the training process and supervision of training participants), content-related supervision (issues related to the need to expand/deepen knowledge about a specific disorder or other training topic), instrumental supervision (issues related to the need to practice specific skills to lead training), navigational supervision (issues related to the need for additional motivation of participants, management of difficult situations, conflict-solving), technical supervision (issues related to the use of digital applications during training and other technical aspects), motivational supervision (questions related to psychological readiness to lead training, burnout and general need for support).

The comparative usability for modules centred on MH (mental health) disorders and newly developed modules centred on mental health crises (aggressive behaviour, self-harm behaviour, suicide and life-threatening behaviour, unusual and disorganized behaviour) was used as the extendability measurement of the UMHT.

### Data analysis

The data analysis was performed using JASP 0.14.3 (GNU Affero GPL v3, open-source license). Descriptive statistics (mean, standard deviation, median and interquartile range, frequency analysis) were used to describe the general results. The nonparametric Kruskal-Wallis one-way ANOVA was used to analyse multiple differences between independent samples, including Dunn`s Post Hoc Test. The Shapiro-Wilk test was used to analyse the normality of distributions. The non-parametric tests were used due to the deviations of the data from the normal distribution.

In this study, we present the means and standard deviations in the text alongside the medians and interquartile ranges in the tables to provide a comprehensive view of the results. While the data are not normally distributed, reporting means and SDs allow for consistency with previous research and provide additional insight into the average values and variability.

Qualitative data analysis was performed using thematic analysis (coding with the following identification of recurring themes and patterns in participants’ responses to open questions). The coding process included familiarisation with data, initial coding, refinement of codes and themes and final thematic structure. Several reliability checks were incorporated: investigator triangulation, reflexivity and participants’ checking.

## Ethics

All participants gave written informed consent to participate in the study. The research team adhered to the Declaration of Helsinki and Model Code of Ethics of the European Federation of Psychologists Associations (EFPA), the Code of Ethics of the National Psychological Association of Ukraine, a member of the EFPA, Inter-Agency Standing Committee (IASC) Recommendations for Conducting Ethical Mental Health and Psychosocial Research in Emergency Settings and British Educational Research Association (BERA) Ethical Guidelines for Educational Research.

The UMHT delivery and data collection protocol was approved by the Ethics Committee of the Zhytomyr Ivan Franko State University (registered in the Office for Human Research Protections), approval number 01–2905/2020 (29 May 2020). The confirmation of compliance with Ukrainian legislation was issued by the Ethics Committee of the National Psychological Association of Ukraine (member of EFPA).

## Results

To assess the programme demand, we measured its actual use in 2021–2023 based on statistics gathered by the MH4U Project, its subcontractor, the NGO ^2^ For Life, responsible for conducting the Trainings of Trainers and trainers’ accreditation and partner organisations supporting the UMHT implementation ( Table 3 and Table 4).

**Table 3.** Statistics on actual use of the UMHT ToTs *.

Years	UMHT ToTs (events / persons)		Donors and supporters
2021	1	35	MH4U Project, Ministry of Health
2022	1	22	
2023	4	113	
	1	19	MH4U Project, Zhytomyr State University
	1	15	German Corporation for International Cooperation
	1 ^3^	30	Friedrich Naumann Foundation for Freedom
	1 ^4^	20	UNDP Turkmenistan, Ashgabat
**Total**	**10**	**254**	6

* ToTs – Trainings of Trainers.

**Table 4.** Statistics on the actual use of the UMHTs.

Years	UMHTs (events / trained persons)		Donors and supporters	Implementers	Region
2021	12	305	MH4U Project	NGO Mental Health Support	Lugansk, Donetsk
	15	291		NGO DeStigma	Lviv
	**27**	**596**	**1**	**2**	**3**
2022	3	68	MH4U Project	NGO Mental Health Support	Lugansk, Donetsk
	5	101		NGO DeStigma	Lviv
	5	96		NGO "Nevermind"	Chernivtsi
	8	142		NGO Mental Health Service	Rivne
	2	60	IREX ^5^, Ministry of Social Affairs, Ministry of Veterans	IREX Ukraine, NGO VeteranHub	Kyiv
	1	30			Vinnytsia
	1	30			Dnipro
	1	25			Uzhhorod
	2	50			Lviv
	1	18			Ternopil
	1	25			Khmelnytskyi
	2	52	Without donor support	NGO Healthy Child Space	Rivne
	1	23		Pidhorodne City Council, NGO Source of Support	Dnipro
	2	59		NGO Here and Now, Bukovinian State Medical University	Chernivtsi
	**35**	**779**	**4**	**10**	**11**
2023 (QI- QIII)	18	170	MH4U Project	NGO Embrace	Kyiv
	10	205		NGO Analytical Platform	Dnipro
	13	181		NGO "Nevermind"	Chernivtsi
	18	365		NGO Space of your possibilities	Rivne
	3	75		Chervonograd Centre of Social Services	Lviv
	3	75	Zhytomyr RMA ^6^, Olena Zelenska Foundation	Zhytomyr State University	Zhytomyr
	2	20	GIZ	NGO Vzaemodia	Zaporizhzhia
	1	10	FLC	NGO Women's Information Consultative Center	Zhytomyr
	9	230	FNF	NGO SmartOsvita	Online for all regions
	1	30	IREX, Ministry of Social Affairs, Ministry of Veterans	IREX Ukraine	Zhytomyr
	1	30			Rivne
	1	30			Poltava
	1	30			Ivano-Frankivsk
	2	20	Without donor support	NGOs New social vector, Ukrainian Institute of Anti- Crisis Management	Kyiv
	1	5		NGO MARTIN-club	Dnipro
	1	11		Red Cross Ukraine	Rivne
	2	20		Department of Education of Zhytomyr City Council	Zhytomyr
	1	11		Community Development Fund "Initiative"	Poltava
	2	30		NGOs Proliska, Life is	Online for all regions
	**90**	**1548**	**9**	**18**	**9 + 2** online for all regions
**Total**	**152**	**2923**			

Within the frame of the original programme, eight training events for trainers were conducted during 2021–2023 (QI-QIII). Among them, six trainings were solely supported by the MH4U Project (170 persons engaged), one in collaboration with a local university in the frame of a master programme (19 persons) and one by the German development agency GIZ (15 persons). Two more trainings for trainers were developed and conducted with a modified version of the programme but with its core 5-step interaction model for Ukrainian teachers (30 persons) and Turkmen MHPSS workers (20 persons).

The UMHT implementation during 2021–2022 covered 15 Ukrainian regions with the biggest numbers of trainees in Lugansk & Donetsk (373), Lviv (517) and Rivne (600). 138 training events were conducted with support from the different donors, while 14 trainings (from data we were able to reach) were run by trainers either voluntarily or with payments from organisations which ordered the training. Altogether, 2923 frontline professionals combined into 152 training groups received skills and knowledge on interaction with people with mental health conditions through the UMHTs. Each year, the number of trained FLPs increased (596 in 2021, 779 in 2022, 1546 in I-III quarters of 2023), as well as the number of donors and supporters (1; 4; 9).

To implement the UMHT in Ukraine, the MH4U Project contracted eight NGOs working in the mental health field during 2021–2023. Besides the MH4U’s disseminating strategy, UMHT was implemented by four NGOs with support from other international donors (IREX ^7^, GIZ ^8^, FNF ^9^, FLC ^10^). Alongside international support, the UMHT and the UMHT-based programmes were funded by the Olena Zelenska Foundation and supported by the Ministry of Health, Ministry of Veterans and Ministry of Social Affairs. Additionally, in the process of the UMHT dissemination, around 10 NGOs, two city councils, and two state universities were engaged.

With the aim to understand the ***acceptability of the UMHT***, we measured the satisfaction with training content and delivery as well as a post-training boost of preparedness to lead the UMHT for its trainers and the preparedness to interact with people with MH conditions for the trained frontline professionals ( Table 5). The detailed descriptive statistics are available in the Extended Data (Table 1–Table 4) ( Gorbunova & Klymchuk, 2024).

**Table 5.** Satisfaction with UMHT content, delivery and preparedness to lead the UMHT (for UMHT trainers) / to interact with people with mental health conditions (for trained FLPs) *.

	UMHT ToT participants				UMHT participants			
	Mean (0 to 5)	SD	Median (0 to 5)	IQR	Mean (0 to 5)	SD	Median (0 to 5)	IQR
*Satisfaction with UMHT content and delivery*								
Training material was structured and clear	4.910	0.311	5	0	4.791	0.511	5	0
Balance between theory and practice was kept	4.806	0.477	5	0	4.769	0.525	5	0
There were enough examples and explanations	4.681	0.563	5	1	4.742	0.561	5	0
Answers to participants’ questions were full and clear	4.701	0.543	5	1	4.739	0.557	5	0
Trainers where careful of sensitive topics and mindful of stigma	4.903	0.297	5	0	4.864	0.423	5	0
Presentations and following material were high quality and perceptibility	4.840	0.453	5	0	4.800	0.503	5	0
Satisfaction mean	**4.807**	**0.291**	**5**	**0**	**4.784**	**0.434**	**5**	**0**
*Preparedness to lead UMHT (for trainers) / to interact with people with mental health conditions (for FLPs)*								
My understanding of the topic of interactions with people with MH issues has increased	4.715	0.550	5	0	4.573	0.652	5	1
My skills to lead UMH trainings and supervisions was mastered / My skills to interact with people with MH issues was mastered	4.410	0.596	4	1	4.366	0.767	5	1
My readiness to lead UMH trainings and supervisions has increased / My readiness to work with people with MH issues has increased	4.597	0.571	5	1	4.429	0.768	5	1
Preparedness mean	**4.574**	**0.438**	**5**	**1**	**4.456**	**0.650**	5	0

* FLPs – Frontline Professionals

Means for general satisfaction with trainings content and delivery is high in both groups (4.807 [0.291] for UMHT trainers; 4.784 [0.434] for trained FLPs) with a bit lower rate for satisfaction with number of examples and explanations (4.681 [0.563]; 4.742 [0.561]) and answers to participants’ questions (4.701 [0.543]; 4.739 [0.557]). Level of the preparedness to perform the duties participants were trained also is high but lower in comparison with training satisfaction (4.574 [0.438], 4.456 [0.650]). Also, in both cases, higher grades received preparedness in terms of knowledge (4.715 [0.550]; 4.573 [0.652]) and lower grades – preparedness in terms of skills (4.410 [0.596]; 4.366 [0.767]).

Regarding knowledge and skills that the FLPs would like to strengthen in order to interact with people with MH conditions, they mentioned the need to practice everything gained during the training in real workplace situations. Also quite common was a desire to dive deeper into mental health topics and have more information *(“learn more psychological stuff”, “know symptoms better”, “to distinguish different kinds of MH conditions”, “how to work with parents of children with MH disorders”*). Personal stress resistance, self-care, resilience, emotional stability, and resourcefulness were also named. There were also several feedbacks with unwillingness, caution or worries toward interaction and support for people with MH issues *(“I am not sure I am ready”, “I am afraid but willing to try”, “I would like to meet less of such cases”*).

Among the skills the UMHT trainers named as those they would like to master more mainly are technical and organisational skills needed to conduct training remotely (e.g., *“use different e-applications”; “trainer skills to organise group work online”, “manage difficult situations online”, “work with resistance and group dynamic without being in one physical space*”) as well as skills required to lead discussion and answer difficult questions ( *“readiness to solve difficult situation”; “communicative skills”, “skills to set limits for discussion”*). As for necessary knowledge, the trainers mentioned a deeper understanding of some MH conditions ( *“epidemiology of MH disorders”, “to renew knowledge about MH conditions I am not used often to work with”, “differential diagnostic of MH disorders”*).

The main suggestions from both surveyed audiences were to have more time to practice and more particular examples; those trained remotely were often asking to switch to the offline training mode.

The averaged data from the accreditation assessment of the UMHT trainers’ knowledge and skills observed in the process of training delivery to the different groups of the FLPs show the lowest means exact for the subject knowledge (3.207 [1.090]) and the highest for the ethical skills (3.915 [0.661]). Table 6 shows generalized results for 41 trainers who have undergone the accreditation process, with only 26 persons passing it successfully with an overall and every-skill grade equal to or above 3.5 ( Table 6). The detailed descriptive statistics are available in the Extended Data (Table 5) ( Gorbunova & Klymchuk, 2024).

**Table 6.** Accreditation assessment of UMHT trainers’ knowledges and skills (0 to 5).

	Subject knowledge	Organisational skills	Instrumental skills	Motivational skills	Ethical skills	Skills & Knowledge means
**Mean**	3.207	3.671	3.720	3.646	3.915	3.632
**SD**	1.090	1.058	0.837	0.785	0.661	0.699
**Median**	3	4	3.5	4	4	4
**IRQ**	2	1	1	1	1	1

With the aim of assessing the ***adaptability of the UMHT*** to the needs of different frontline professionals, we compared the usability of the programme by different professional groups ( Table 7). The highest generalized usability means have educators (0.676 [0.118]), police officers (0.669 [0.098]) and social workers (0.663 [0.113]); lowest – librarians & museum workers (0.578 [0.127]) and emergency responders (0.580 [0.134]). Post Hoc Dunn tests for the same generalized data show significant differences for the mentioned groups with high-low usability means: educators and emergency responders (4.709 [p < 0.001]); educators and librarians & museum workers (4.031 [p < 0.001]; police officers and emergency responders (4.166 [p < 0.001]); police officers and librarians & museum workers (3.631 [p < 0.001]); social workers and emergency responders (4.121 [p < 0.001]); social workers and librarians & museum workers (3.533 [p < 0.001]). The detailed descriptive statistics are available in the Extended Data, Table 6 ( Gorbunova & Klymchuk, 2024).

**Table 7.** Usability of the UMHT by different groups of the FLPs *.

	educators	emergency responders	librarians & museum workers	military volunteers	pharmacists	police officers	priests and clerics	social workers	employees of occupation centres
Valid	152	52	29	34	37	122	27	203	58
Recognise (0-4)									
Median	4	4	4	4	4	4	4	4	4
Mean	3.678	3.365	3.414	3.735	3.649	3.787	3.889	3.734	3.603
Std. Deviation	0.667	1.067	1.053	0.567	0.588	0.564	0.424	0.723	0.897
IQR	0	1	1	0	1	0	0	0	0
Validate (0-4)									
Median	4	4	4	4	4	4	4	4	4
Mean	3.368	3.269	3.103	3.706	3.622	3.475	3.741	3.488	3.276
Std. Deviation	0.94	1.254	1.372	0.462	0.758	1.006	0.859	1.036	1.089
IQR	1	1	2	1	0	0	0	0	1.75
Support (0-5)									
Median	3	3	2	3	3	3	3	3	3
Mean	2.842	2.5	2.517	2.559	2.865	2.836	2.852	2.631	2.586
Std. Deviation	1.08	1.076	0.911	0.927	1.134	1.153	0.949	1.032	0.838
IQR	2	1	1	1	1	2	1.5	1	1
Refer (0-3)									
Median	1	1	1	1	1	1	1	1	1
Mean	1.362	1.135	1.172	1.118	1.243	1.254	0.926	1.3	1.241
Std. Deviation	0.858	0.817	0.889	0.729	0.83	0.777	0.675	0.779	0.885
IQR	1	1	1	0.75	1	1	0	1	1
Ensure (0-3)									
Median	2	1	1	2	1	2	2	2	1
Mean	1.789	1.096	1.103	1.588	1.162	1.631	1.519	1.655	1.466
Std. Deviation	0.925	0.774	0.673	0.743	0.958	0.73	0.849	0.789	0.842
IQR	2	1	0	1	2	1	1	1	1
Total (0-19)									
Median	13	11.5	11	13	12	13	13	13	12
Mean	13.039	11.365	11.31	12.706	12.541	12.984	12.926	12.808	12.172
Std. Deviation	2.165	2.582	2.466	1.586	1.804	1.876	1.591	2.157	1.874
IQR	3	3	3	2	2	2	2	2	3
Did you work with people with mental disorders after the UMHT? (0/1)									
Median	1	1	1	1	1	1	1	1	1
IQR	0	0	0	0	0	0	0	0	0
Mean	0.98	0.904	0.862	1	1	0.975	1	0.966	0.983
Std. Deviation	0.14	0.298	0.351	0	0	0.156	0	0.183	0.131
%	98%	90%	86%	100%	100%	98%	100%	97%	98%
95% CI (lower limit)	96%	82%	74%	100%	100%	95%	100%	94%	95%
95% CI (upper limit)	100%	98%	99%	100%	100%	100%	100%	99%	100%
Did you use the knowledge and skills gained during the UMHT? (0/1)									
Median	1	1	1	1	1	1	1	1	1
IQR	0	0	0	0	0	0	0	0	0
Mean	0.967	0.942	0.966	1	1	0.992	1	0.99	0.983
Std. Deviation	0.179	0.235	0.186	0	0	0.091	0	0.099	0.131
%	97%	94%	97%	100%	100%	99%	100%	99%	98%
95% CI (lower limit)	94%	88%	90%	100%	100%	98%	100%	98%	95%
95% CI (upper limit)	100%	100%	100%	100%	100%	100%	100%	100%	100%

* FLPs – Frontline Professionals

As for the separate groups of FLPs, the biggest number of significant intergroup differences according to ANOVA and Dunn`s Post Hoc tests is observed for the question *“Did you work with people with mental disorders after the UMHT?”*. The lowest means are attributed to librarians & museum workers (0.862 [SD=0.351]). The intergroupe differences between them and other FLPs are as follows: educators (-3.301 [p < 0.001]), military volunteers (-3.088 [p = 0.002]), pharmacists (-3.148 [p = 0.002]), police officers (-3.105 [p = 0.002]), priests & clerics (-2.919 [p = 0.004]), social workers (-2.949 [p = 0.003]) and employees of occupation centres (-3.003 [p = 0.003]).

As for the self-assessed ability to recognize MH condition, Post Hoc Dunn tests show significant differences between emergency responders and such groups as social workers (-3,382 [p < 0.001]), police officers (-3,151 [p = 0.002]) and priests & clerics (-3,059 [p = 0.002]). The most significant differences in the self-assessed abilities to validate MH condition with a person and to refer for professional help were found between educators and priests & clerics (-2,645 [p = 0.008]). As for the self-assessed ability to ensure that professional help was reached there are significant differences between educators and such FLPs as emergency responders (4.982 [p < 0.001]), librarians & museum workers (4.080 [p < 0.001]), pharmacists (3.719 [p < 0.001]); emergency responders and military volunteers (-2.687 [p = 0.007]), police officers (-3.788 [p < 0.001]), social workers (-4.306 [p < 0.001]) and finally police officers and librarians & museum workers (3.165 [p = 0.002]).

Considering averages ( Table 7), less used among all the groups are the abilities to refer and be sure that professional help was reached. The detailed results of the ANOVA and Dunn's Post Hoc tests for all variables are available in Tables 7–22 in the Extended Data ( Gorbunova & Klymchuk, 2024).

**Figure 1.**
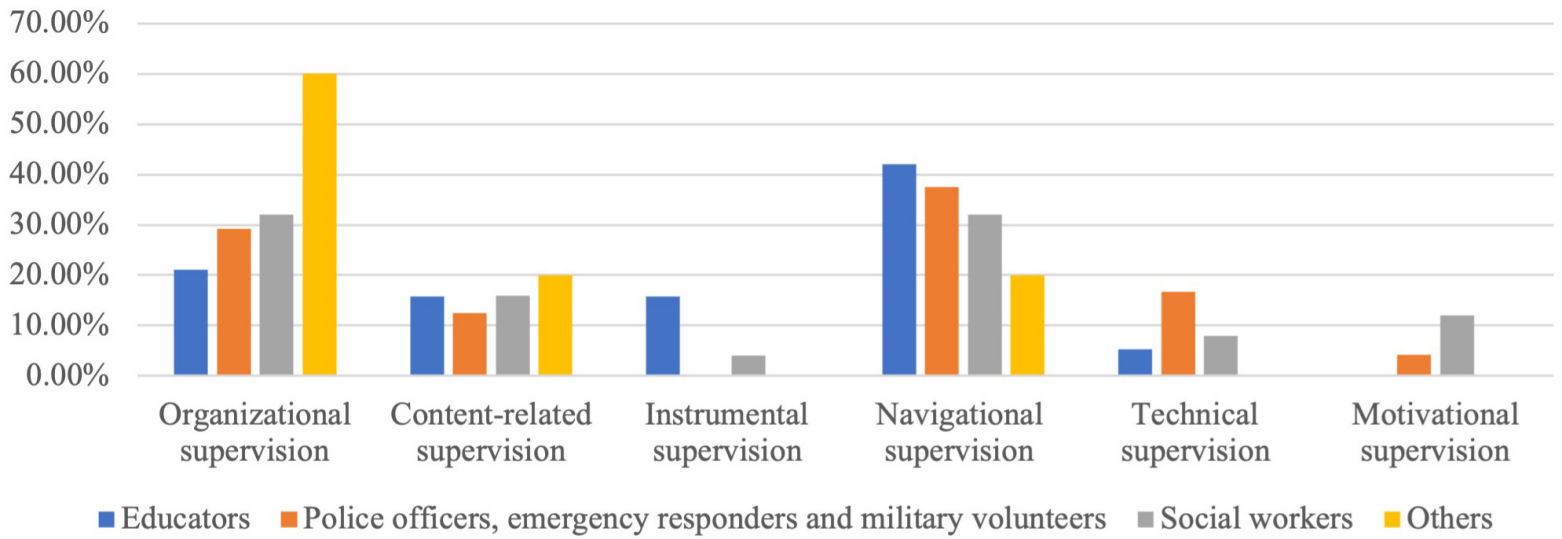
Supervision reports analysis.

The most frequently requested supervisions were navigational (35.62 %) and organisational (30.14 %), related to participants’ motivation, conflict resolution, and the organisation of the training process ( Figure 1). The data were driven from supervisors’ reports on 73 supervisions of the UMHT trainers concerning their work with educators (19 groups), police officers, emergency responders & military volunteers (24), social workers (25) and other FLPs (employees of occupation centres (2), librarians & museum workers (1), priests & clerics (1), and pharmacists (5).

The ***extendability*** of the UMHT was measured through a comparison of programme usability by FLPs who were trained and then worked with only disorder-centred modules (636 persons), only with the newly developed modules for the interaction with people with four different mental health crisis behaviours (63) and with both modules, disorders and crises-centred (15) ( Table 8).

**Table 8.** Usability of UMHT with different combination of the training modules.

	Crisis	Disorders	Crises & Disorders
Valid	63	636	15
Recognise (0-4)			
Median	4	4	4
Mean	3.81	3.667	3.8
Std. Deviation	0.396	0.763	0.561
IQR	0	0	0
Validate (0-4)			
Median	4	4	4
Mean	3.714	3.417	3.2
Std. Deviation	0.682	1.034	1.207
IQR	0	1	2
Support (0-5)			
Median	3	3	3
Mean	2.73	2.712	2.533
Std. Deviation	1.139	1.044	0.915
IQR	2	1	1
Refer (0-3)			
Median	1	1	1
Mean	1.365	1.245	1.333
Std. Deviation	0.679	0.821	0.9
IQR	1	1	1
Ensure (0-3)			
Median	2	2	2
Mean	1.603	1.563	1.6
Std. Deviation	0.73	0.861	0.507
IQR	1	1	1
Total (0-19)			
Median	13	13	13
Mean	13.222	12.604	12.467
Std. Deviation	1.66	2.158	2.588
IQR	2.5	3	4
Did you work with people with mental disorders after the UMHT? (0/1)			
Median	1	1	1
IQR	0	0	0
Mean	1	0.965	0.933
Std. Deviation	0	0.183	0.258
%	100%	97%	93%
95% CI (lower limit)	100%	95%	81%
95% CI (upper limit)	100%	98%	100%
Did you use the knowledge and skills gained during the UMHT? (0/1)			
Median	1	1	1
IQR	0	0	0
Mean	1	0.98	1
Std. Deviation	0	0.142	0
%	100%	98%	100%
95% CI (lower limit)	100%	97%	100%
95% CI (upper limit)	100%	99%	100%

According to the obtained data, there is only one significant intergroup difference between FLPs who studied only disorders and only crises for validation MH conditions (Post Hoc Dunn tests 2,185 [p = 0.029]). Detailed descriptive statistics are available in the Extended Data, Table 23 ( Gorbunova & Klymchuk, 2024). The detailed results of the ANOVA and Dunn`s Post Hoc tests for all variables are available in Tables 24–38 in the Extended Data ( Gorbunova & Klymchuk, 2024).

## Discussion

### Demand: to what extent is the UMHT likely to be used?

Numbers on the actual use of the UMHT for different groups of FLPs are direct evidence of its demand. Based on reachable data, during 2021–2023 were conducted 152 UMHTs with 2923 persons involved. Every year, the number of events, trained FLPs, donors & supporters and implementers (2, 10, 18) grows. Despite decreased training events in the first half-year of 2022 because of the full-scale Russian invasion, demand for the UMHT stabilised and increased. The UMHT dissemination has gained support from new international donors and community-based stakeholders such as city councils, local NGOs, and universities. The Zhytomyr State University has even included the UMHT in the learning curriculum for the Master's Programme in Public Mental Health.

The demand for public mental health prevention programmes is rarely assessed, mainly because of their on-demand development, when a programme is an answer to population needs.

For example, the PFA as a crisis response approach was developed in the so-called post-9/11 era as a quick and effective tool to reduce trauma-related stress, and shortly after, it was included in international treatment guidelines for PTSD and as an early intervention and adopted by such humanitarian organisations as International Federation of Red Cross and Red Crescent Societies ( Shultz & Forbes, 2014). There is only a few feasibility research on the FPA demand, for example, among police officers exposed to traumatic events and healthcare workers during the COVID-19 pandemic, where demand was estimated through the programme's capacity to meet the needs of its beneficiaries based on participants' interviews in the first case and on the expression of interest to participate in the programme in the second ( Chandler *et al.*, 2023; Geoffrion *et al.*, 2023).

The MHFA started as a workplace-focused intervention to help depressed employees and, in the first place, was run voluntarily by its developers as a service to the local community. The programme evolved into a global movement with a well-established delivery system with 30 licensed providers in 29 countries supported by politicians and celebrities ( Kitchener & Jorm, 2008).

The mhGAP Programme was launched, promoted and implemented with the support of WHO regional offices in over 100 countries in response to the gap between the available and needed resources to address the burden of mental disorders in low- and middle-income countries ( Hughes & Thomson, 2019; Mills & Hilberg, 2019). The programme follows a detailed guide for implementation, starting with establishing the implementation team and ending with a thorough monitoring and evaluation process ( Spagnolo & Lal, 2021).

The UMHT was also developed on demand by a number of stakeholders on different levels, including the Ukrainian ministries of Health, Veterans Affairs, and Social Policy.

The Ministry of Health of Ukraine approved the UMHT and added institutional certification for ToT trainers. The Ministry of Veterans includes the UMHT in the Recommendation guide for the local authorities, "Psychosocial Assistance to War Veterans and Members of their Families" ( Ministry of Veterans Affairs of Ukraine, 2021).

The UMHT 5-step model became a base for the IREX Ukraine Veteran Reintegration Programme "Strengthening the Capacity of Social Workers to Provide Mental Health Support for Veterans and their Families," which was implemented in cooperation with the Ministry of Social Policy of Ukraine. The manual for this programme has been viewed over 60,000 times online and requested by over 650 social workers, psychologists, and teachers ( IREX, 2022). The biggest Ukrainian NGO, SmartOsvita, working in the field of school education, launched an UMHT-based training programme, “Psychosocial Support for Educators” ^11^, with the plan to engage around 1000 schoolteachers by the end of 2023.

One more adaptation of the UMHT 5-step model was done at the request of the UNDP Turkmenistan office to prepare local trainers in the context of MHPSS and peacebuilding.

Such support from different players, as well as adaptation and dissemination even beyond the primary schema, are sufficient evidence of the programme’s demand.

### Acceptability: to what extent is the UMHT suitable, satisfying and attractive to programme deliverers and recipients?

To answer the research question of acceptability, we surveyed two main audiences: programme trainers as its deliverers and frontline professionals as its recipients. In both cases satisfaction with training content and delivery is highly sufficient. It is essential to mention that every satisfaction measurement remained stable in the transition from the UMHT trainers to trained FLPs; this speaks in favour of the proposed ToT model and supervision.

However, either UMHT trainers and FLPs would like a bit more clarity and completeness from trainers’ side in answers to their questions and suggest paying more time and attention to practice and examples. The issue with perceived lack of practice and due to it, needed skills is also reflected in the scoring of personal preparedness to perform expected duties after training. Participants rated their understanding of the topic above their skills in leading UMH training and supervising trainees.

It is a typical case when knowledge is the first takeaway from skills-oriented training. For example, in the feasibility assessment of FMHA for indigenous peoples in Canada, participants emphasised an increase in mental health knowledge as one of the most valuable impacts from the training. Similarly, the mhGAP feasibility study in India revealed a significant increase in the mental health knowledge of healthcare workers, which stood out as a key post-training result ( Crooks *et al.*, 2018; Siddaiah *et al.*, 2022). It is natural for practical skills to lag slightly knowledge and general awareness, even after well-balanced training. People need first apply gained skills in real work-life situations. Additionally, they need to feel resourceful, resilient, and stable to effectively work with mental health topics. To support our UMHT trainers and frontline professionals from the beginning, obligatory after-training supervision with the possibility of address any, even personal, issues connected with the UMHT was implemented. Detailed instructions on adapting the organisation process and every training exercise to remote mode were developed and placed in the UMHT Trainer’s Manual from the start of the programme piloting to help the UMHT trainers be more confident while conducting online training with FLPs, ( MH4U, 2023). During supervision, each trainer could consult with the supervisor on using e-applications and other useful tools for e-learning facilitation. Supervision support was also offered to fill in knowledge gaps of some mental health conditions.

To maintain the quality of training delivery, an accreditation procedure was implemented as permission to enter practice as a UMHT trainer. To pass through accreditation, according to the programme’s requirements, a trainer should score no less than 3.5 in subject knowledge (how well a trainer knows the training material, is fluent in the topics, and gives correct and comprehensive answers to the questions of the participants); organisational skills (how effectively trainer organises the process, keeps the timing); instrumental skills (how effectively trainer uses and applies training tools and techniques); motivational skills (how successfully trainer motivates the participants and how effectively works with resistance, complaints and other potential conflicts); and ethical skills (how ethical are trainer’s manner and behaviour in terms of supporting the dignity of the participants and being free of stigma and bias toward mental health, gender, age, profession *etc.*).

### Adaptability: to what extent is the UMHT suitable for adjusting content and procedure to new formats and working with different population groups?

The main evidence of the UMHT's adaptability is the high marks for its usability and more or less even distribution of trained skills utilisation among different groups of FLPs.

The between-group differences (see Extended Data, Table 6) ( Gorbunova & Klymchuk, 2024) are well explained by specifics of the FLPs’ professional duties as well as the possibilities and limitations of their daily work. For example, the highest rates of the usability of all UMHT skills are reported by educators, police officers and social workers as professionals who have not just one occasional encounter but chances to return and see their clients again. Their answers differ from librarians & museum workers, and emergency responders whose self-assessed rates of programme usability are the lowest, The thinkable reasons for such a difference are a lower level of stress people experience visiting a library or museum that smoothens their behaviour in the first case and lack of time for interaction in the second. That is possibly why librarians and museum workers have less reason to speak about mental health despite having time to do so, while emergency responders, on the contrary, lack time to interact with people; however, they can see a lot of signs of mental difficulties. In addition to the lower average usability, data on librarians and museum workers shows that they do not meet people with MH difficulties as often as other FLPs, especially educators, police officers, social workers, military volunteers, pharmacists, priests & clerics and employees of occupation centres.

There are intergroup differences between the separate skills. Thus, the ability to recognise MH condition is less usable by emergency responders who servantly have less time and space to observe people's reactions and behaviour in comparison with social workers, police officers and priests & clerics. The ability to validate MH condition is most usable by priests & clerics who, because of their duties, sort of, are permitted to speak about mental health; they significantly differ from educators who can be perceived by students and caregivers as not right persons to share mental difficulties with. At the same time, educators are the ones who, most often in comparison with the majority of other studied FLPs, keep track if the referral was successful, probably because they are able to see and check their students on a regular basis.

Analysis of supervision requests from the UMHT trainers shows that the FLPs, first of all, need additional motivation to support people with MH conditions and skills to smooth interaction and prevent possible conflicts. Keeping in mind that among the skills that FLPs would like to strengthen, they often mention those connected with resilience that are not directly included in the UMHT, it is essential to have supervisor support as a mandatory part of the UMHT implementation.

Taking into account the similarity (but not sameness) of the skills trained in the UMHT and the MHFA programmes, it is possible to compare their usability from the side of people who intend to help others. Thus, the MHFA feasibility trial in the workplace in the UK shows high responses to the programme in higher education, construction/engineering and health sectors ( Crooks *et al.*, 2018). One more thing to distinguish is the trained audience. The FMHA is developed for peer-to-peer usage in the workplace when the UMHT trains additional skills that are not necessarily required for professional interaction between service providers ( *i.e.*, FLPs) and users.

As for mental health support as the partial duty of such service providers as educators, police officers, social workers and other frontline professionals, there is data on the existence of such public and governmental requests in different countries. Still, there is a lack of adequate preparedness to manage people with mental health needs ( Aviram, 2002; Jain *et al.*, 2021; Lamb *et al.*, 2002; Mazzer & Rickwood, 2015; Mclean & Marshall, 2010; Rothì *et al.*, 2008). In all cases, the authors emphasise the need to develop special educational programmes tailored to the MH support deliverers' needs. The UMHT proposes a three-step scheme of such tailoring, which enhances programme adaptability. The first is the analysis of the needs of a target audience ( *i.e.*, a group of FLPs), the second is the needs-adjusted training delivery, and the third is the obligatory supervision support.

### Extendability: to what extent can the UMHT be expanded to cover new topics and solve new problems?

The idea of the UMHT extendability was one of the baselines at the stage of the programme development. The training is called universal because of its universal 5-step model that offers a standard frame for interaction with people with mental health conditions and is suitable for different types of frontline professionals. This frame allows the training curriculum to add new modules dedicated to new disorders as any mental health-connected issue. The UMHT steps first imply recognition of MH condition or any connected problem (paying attention, forming hypothesis, getting ready, preparing space), second, validating of MH condition with a person or caregiver (establishing contact, building trust, testing readiness to speak about MH, finding out the awareness), third, supporting a person/caregiver (sharing observations, decreasing stigma, giving simple advice, responding with special techniques, bringing hope), fourth, referring for professional help (describing possibilities of professional help, naming non-evidence based approaches, helping to contact a professional), fifth, ensuring the reference was successful (initiating next meeting, learning more about person’s MH, helping to contact a professional if the first reference did not work).

The research team had a chance to test the extendability of the training in 2022 when two departments of the National Policy of Ukraine in Lugansk and Donetsk oblasts (regions whose territories are currently occupied by Russia or are proximately close to the battlefield) requested to include in the UMHT modules on mental health crisis and adjust the 5-step model to reaction on people behaviours (aggressive behaviour, self-harm behaviour, suicide and unusual & disorganised).

The comparative analysis of the usability data collected for the training groups that studied MH disorders, disorders, and crises and solely crises show no significant intergroup differences. Only statistically proven differences were seen between FLPs who studied only disorders and only crises for validation MH conditions. This result can be explained by partial redistribution of skills application in such steps as validation and support. In case of mental crisis, it is often an issue that support, at least responding with special techniques, comes and is used first before or even without condition validation.

## Limitations and future research

The main limitation of the performed feasibility research is the absence of effectiveness data on UMHT’s impact on recipients’ mental health and related outcomes. The authors are conscious of this limitation, especially in the context of the recently published Cochrane review on the methodologically closest programme, MHFA ( Richardson *et al.*, 2023). The next steps of the programme’s evaluation require an analysis of referrals for professional help and then scheduled appointments together with mental health monitoring as, for example, in the case of the FPA programme implemented for healthcare workers in Arizona, USA, during the COVID-19 Pandemic or the mhGAP feasibility analysis done in Makueni County, Nepal and Nigeria ( Chandler *et al.*, 2023; Mutiso *et al.*, 2019a; Salisbury *et al.*, 2021).

Additional limitations include potential threats to internal validity from self-reporting measures, limits of external validity based on participants' characteristics, and a lack of reliability and validity information for the measures used. Due to the specific study context, the findings may not generalise beyond the socio-political context of Ukraine. In the future, research consideration will be given to more objective measures, e.g. supervisors’ assessments and role-play structural observation and exploring the long-term effects of the UMHT. The mitigation measures regarding selection bias and reliability/validity of the self-reporting tools will be explored and implemented.

## Conclusion

The feasibility of public mental health interventions is one of the primary requirements for their implementation, especially in countries with low and middle incomes where resources are limited. The UMHT as an educational programme for frontline professionals was developed and piloted in Ukraine in 2021–2023 to support the country's transition from highly institutionalised to community-based MH care when an intersectional collaboration and sustained referral system are crucial.

Assessment of the UMHT feasibility based on its actual use, users’ satisfaction, usability for different groups of frontline professionals, and openness to modifications show the programme’s potential for further development and implementation.

During 2021–2023, even with obstacles connected with the full-scale Russian invasion in Ukraine, 2923 frontline professionals passed the UMHT. Every year, the number of events, trained persons and engaged implementers increased. The UMHT 5-step model became a base for several other public mental health promotional & preventional interventions.

Programme trainers as its deliverers and frontline professionals as its recipients reported high satisfaction with the training content and delivery as well as preparedness to apply their gained knowledge and skills in practice.

The main evidence of the UMHT's adaptability and extendability is the high scores for its usability for different groups of frontline professionals and for different mental conditions. Besides, the 5-step model laid in the programme's base offers frontline professionals a universal frame of interaction with people with MH conditions and is suitable for different types of mental health difficulties.

## Ethics and consent

All participants gave written informed consent to participate in the study. The research team adhered to the Declaration of Helsinki and Model Code of Ethics of the European Federation of Psychologists Associations (EFPA), the Code of Ethics of the National Psychological Association of Ukraine, a member of the EFPA, Inter-Agency Standing Committee (IASC) Recommendations for Conducting Ethical Mental Health and Psychosocial Research in Emergency Settings and British Educational Research Association (BERA) Ethical Guidelines for Educational Research.

The UMHT delivery and data collection protocol was approved by the Ethics Committee of the Zhytomyr Ivan Franko State University (registered in the Office for Human Research Protections), approval number 01–2905/2020 (29 May 2020). The confirmation of compliance with Ukrainian legislation was issued by the Ethics Committee of the National Psychological Association of Ukraine (member of EFPA).

## Data Availability

Zenodo: Universal mental health training for frontline professionals (UMHT)'s feasibility analysis. https://zenodo.org/records/10926232 ( Gorbunova & Klymchuk, 2024). The project contains the following underlying data: UMHT_data_full set_ZENODO_v.2.xlsx Zenodo: Universal mental health training for frontline professionals (UMHT)'s feasibility analysis. https://zenodo.org/records/10926232 ( Gorbunova & Klymchuk, 2024). The project contains the following extended data: UMHT Feasibility Extended Data.pdf UMHT Surveys.pdf Data are available under the terms of the Creative Commons Zero "No rights reserved" data waiver (CC0 1.0 Public domain dedication).
