# Supplementary material for: Universal mental health training for frontline professionals (UMHT)’s feasibility analysis
Source: Open Res Eur. 2025 Mar 21;4:96. Originally published 2024 Apr 29. [Version 3] doi: 10.12688/openreseurope.17358.3 (PMC11883208; doi:10.12688/openreseurope.17358.3)
Supplement: Peer review history

# Reviewer report: Reviewer response for version 3

**DOI:** 10.21956/openreseurope.21661.r52324

**Published:** 2025-04-17

**Authors:** Kathryn E. Kanzler, Chase Aycock

**Affiliations:** 1 37th Human Performance Squadron, US Air Force, Joint Base San Antonio-Lackland, San Antonio, TX, USA; 2 Baylor College of Medicine, Houston, TX, USA

The authors were highly responsive to feedback and the manuscript will be an excellent contribution to the field. We only have 2 final recommendations.

 Re: Tables 5, 6, 7 & 8, the anchors and direction of the responses are unclear. It would help improve the interpretability of findings if these could be added somewhere in the manuscript or in the tables (i.e., what are the Likert scales and dichotomous response options?)

 Re: Table 8, for the last two items, recommend deleting Median, IQR, Mean, St Dev as they are not as relevant for dichotomous responses.

Is the study design appropriate and does the work have academic merit?

Yes

Is the work clearly and accurately presented and does it cite the current literature?

Partly

If applicable, is the statistical analysis and its interpretation appropriate?

Partly

Are all the source data underlying the results available to ensure full reproducibility?

Yes

Are the conclusions drawn adequately supported by the results?

Partly

Are sufficient details of methods and analysis provided to allow replication by others?

Partly

Reviewer Expertise:

clinical health psychology; task-shifting/task-sharing; implementation science

We confirm that we have read this submission and believe that we have an appropriate level of expertise to confirm that it is of an acceptable scientific standard.

# Reviewer report: Reviewer response for version 2

**DOI:** 10.21956/openreseurope.20936.r51283

**Published:** 2025-03-05

**Authors:** Rodolfo Buselli, Antonello Veltri

**Affiliations:** 1 Occupational Medicine Unit, Azienda Ospedaliero Universitaria Pisana (Ringgold ID: 9257), Pisa, Tuscany, Italy; 2 Center for Work-Related Stress and Occupational Mental Disorders, Azienda Ospedaliero Universitaria Pisana (Ringgold ID: 9257), Pisa, Tuscany, Italy; 3 Occupational Health Department, Azienda Ospedaliero Universitaria Pisana, Pisa, Italy; 4 Center for Work-Related Stress and Occupational Mental Disorder, Azienda Ospedaliero Universitaria Pisana (Ringgold ID: 9257), Pisa, Tuscany, Italy

This study provides an important and timely evaluation of the feasibility of the Universal Mental Health Training for Frontline Professionals (UMHT) in Ukraine, a country experiencing significant mental health challenges due to ongoing conflict and social instability. The research adopts a robust mixed-methods approach, combining both quantitative and qualitative data, which adds depth to the analysis. The results show high levels of satisfaction and usability among frontline professionals. The study demonstrates how the training model can be adapted and expanded. Moreover, ethical considerations are appropriately addressed, ensuring the integrity of the research process. However, despite these strengths, there are some limitations that warrant attention and that should be addressed in future studies.

  

- One of the most significant weaknesses of the study is the lack of effectiveness data. While the research confirms that the UMHT is feasible, it does not show whether it actually improves mental health outcomes for the people served by the  trained professionals. Without data on patient outcomes, referral rates, or clinical improvements, it is difficult to measure the true impact of the program beyond participants’ subjective perceptions. The study would greatly benefit from a longitudinal component that tracks the long-term retention and practical application of the skills acquired by the frontline professionals.
- Another issue is the reliance on self-reported data. Although surveys on satisfaction and preparedness provide useful insights into participants’ experiences, they are prone to bias. Participants might overestimate their abilities or give positive responses due to social expectations. More objective methods, such as supervisor evaluations, standardized role-playing exercises, or direct observations, would provide a more reliable assessment of the training’s efficacy.
- The study’s external validity is also a potential concern. Since the research is conducted within the specific socio-political context of Ukraine, it is unclear whether the training would be equally successful in other countries with different cultural and financial constraints. Further research should explore the applicability of the program in diverse settings to determine its broader generalizability.
- Additionally, while the study includes qualitative data, the methods used to analyze participant feedback are not fully explained. The paper refers to participants’ suggestions and comments but does not describe how themes were identified or validated. A clearer explanation of the qualitative analysis process, including coding methods and reliability checks, would improve the transparency and rigor of the study.
- Another limitation is the incomplete reporting of response rates. The study does not clearly state how many participants completed the surveys compared to how many started the program. Without this information, it is difficult to know whether the reported satisfaction levels accurately represent all trained professionals or just a selected group.

Although the article briefly acknowledges some limitations, the discussion on potential threats to validity  could be expanded. For example, the authors briefly mention the lack of effectiveness data but do not thoroughly address potential biases introduced by self-reporting, selection bias, or measurement limitations. A more comprehensive discussion of these methodological challenges and the steps taken to mitigate them would enhance the credibility of the findings.

 In conclusion, this study provides valuable insights into the feasibility of mental health training programs in crisis-affected settings. Its strong methodology and policy relevance make it a strong candidate for publication. However, to maximize its impact and contribution to the field, the study would benefit from a more extensive discussion of its limitations, the inclusion of objective outcome measures, and a plan for future research that assesses the long-term effectiveness of the UMHT. Addressing these issues would enhance the validity of the findings and strengthen the case for incorporating the training program into larger mental health systems.

Is the study design appropriate and does the work have academic merit?

Yes

Is the work clearly and accurately presented and does it cite the current literature?

Yes

If applicable, is the statistical analysis and its interpretation appropriate?

Yes

Are all the source data underlying the results available to ensure full reproducibility?

Yes

Are the conclusions drawn adequately supported by the results?

Yes

Are sufficient details of methods and analysis provided to allow replication by others?

Yes

Reviewer Expertise:

Mental Health Prevention at Work -  Psychosocial Risk Management

We confirm that we have read this submission and believe that we have an appropriate level of expertise to confirm that it is of an acceptable scientific standard.

# Reviewer report: Reviewer response for version 2

**DOI:** 10.21956/openreseurope.20936.r49802

**Published:** 2025-01-30

**Authors:** Kathryn E. Kanzler, Chase Aycock

**Affiliations:** 1 37th Human Performance Squadron, US Air Force, Joint Base San Antonio-Lackland, San Antonio, TX, USA; 2 Baylor College of Medicine, Houston, TX, USA

The authors were highly responsive to feedback and the manuscript appears much stronger now. Please consider these remaining recommendations: 

 Abstract:

- The statement, “All measurements of feasibility analyses usually fall into two broad categories …” sounds definitive, but the citation that was provided does not seem to clearly back up this assertion ( Pfledderer *et al.*, 2024). We recommend deleting the word “All” for accuracy. 

Plain language summary:

- It is unclear why the program is written as “UNMHT” here instead of “UMHT” throughout the manuscript. It seems to be an error. 

Method:

- P. 4, 2 ^nd^ paragraph: What was the rate of participation in the surveys? (Did anyone who received the surveys choose not to participate?) Did 100% of participants respond? If so, then that would be helpful to clarify.

Results:

- Table 8 (and elsewhere as relevant) – Please report the percentage of respondents who answered “yes” or “no” here and elsewhere. It is more interpretable to present percentages (perhaps with 95% CIs) for dichotomous outcomes—e.g., “Did you work with people with mental disorders after the UMHT? (0/1)”

Is the study design appropriate and does the work have academic merit?

Yes

Is the work clearly and accurately presented and does it cite the current literature?

Partly

If applicable, is the statistical analysis and its interpretation appropriate?

Partly

Are all the source data underlying the results available to ensure full reproducibility?

Yes

Are the conclusions drawn adequately supported by the results?

Partly

Are sufficient details of methods and analysis provided to allow replication by others?

Partly

Reviewer Expertise:

clinical health psychology; task-shifting/task-sharing; implementation science

We confirm that we have read this submission and believe that we have an appropriate level of expertise to confirm that it is of an acceptable scientific standard, however we have significant reservations, as outlined above.

# Response

**Published:** 2025-03-20

**Authors:** Viktoriia Gorbunova

**Affiliations:** Department of Behavioural and Cognitive Sciences, University of Luxembourg, Esch-sur-Alzette, Luxembourg District, Luxembourg

Thank you for your valuable feedback and suggestions. We have revised and updated everything accordingly.

# Reviewer report: Reviewer response for version 1

**DOI:** 10.21956/openreseurope.18761.r44657

**Published:** 2024-10-22

**Authors:** Kathryn E. Kanzler, Chase Aycock

**Affiliations:** 1 Baylor College of Medicine, Houston, TX, USA; 2 37th Human Performance Squadron, US Air Force, Joint Base San Antonio-Lackland, San Antonio, TX, USA

The authors are commended for evaluating the feasibility of the Universal mental health training for frontline professionals (UMHT) in Ukraine. This is important information that could impact program implementation elsewhere and stimulate additional research on UMHT and similar programs. Please consider our feedback to further strengthen this paper:

 ABSTRACT

- Results: It would be helpful to add additional context to the numbers in this section (e.g., adding “ *n *=”, “ *p *=”, etc., as appropriate):
  - The range of ratings is needed to interpret the results regarding satisfaction, preparedness and usability (e.g., satisfaction was rated 4.81 on what scale?)
  - Clarification is needed regarding what the parenthetical numbers refer to.
  - The final sentence of the results section also needs explanation because “rates of usability” suggest these are a percentage, but it’s unclear.

PLAIN LANGUAGE SUMMARY

- There is a typo where “UMHT” is written as “UNMHT.”

INTRODUCTION

- p.4, 3 ^rd^ paragraph: a citation is needed for the last sentence, asserting that “all measurements…fall into two broad categories.”
- P. 4, 6 ^th^ paragraph: Related, was an implementation framework or resource used to identify the components of the research questions (demand, acceptability, adaptability, extendibility)? If not, this would be helpful to mention in the Discussion section, because there are frameworks that could be useful in future research (e.g., RE-AIM [Glasgow et al., 2019]).

METHODS

 UMHT dissemination and participants

- P. 4, 1 ^st^ paragraph: Please provide more information about the UMHT training (e.g., Length of time/duration, etc)
- P. 4, 2 ^nd^ paragraph: What was the rate of participation in the surveys? (Did anyone who received the surveys choose not to participate?)
- P. 4, 3 ^rd^ paragraph: More information regarding the methods for conducting preliminary interviews is needed.

Research methods and measurements

- P. 6, 1 ^st^ paragraph: What do the anchor numbers on the 1-5 scale mean? What was “1” and what was “5”?

Data analysis

- It would be helpful to clarify why certain analytic decisions were made (e.g., why nonparametric statistics were used) and provide citations that support those decisions.
- Nonparametric statistics are typically chosen because the data were not normally distributed or because single-item Likert-type questions were used. This indicates that a median and interquartile range (IQR) would be more appropriate for descriptive statistics than mean and standard deviation. Considering this, please consider using medians and IQR for tables 5, 6, 7, 8, and elsewhere where means and SDs are reported. This may affect some of the conclusions drawn.
- How were the qualitative data analyzed? It is unclear how these data were analyzed to generate the themes that are presented in the results sections.

RESULTS

- P. 9, 5 ^th^ paragraph: How were FLP’s desired knowledge and skills assessed? Were these responses from free text questions or interviews?   
- P. 9, 5 ^th^ paragraph - p. 10, 1 ^st^ paragraph: Consider adding a table of identified qualitative themes with relevant quotes to better organize and share these findings
- P. 10, 2 ^nd^ paragraph: Please explain “offline format”
- P. 10, 5 ^th^ paragraph (“As for the separate..”): This paragraph is one long sentence and difficult to understand. Recommend re-phrasing and breaking into shorter sentences.
- P. 10, 6 ^th^ paragraph: please highlight that the respondents rated their own abilities (i.e.,  “As for the self-rated ability to recognize MH condition…” and “self-rated ability to ensure professional help was reached *…"*)
- P. 10, 7 ^th^ paragraph: the first sentence (starting with “considering averages…”) is difficult to understand; please rephrase for clarity
- P. 10, 8 ^th^ paragraph: it is unclear what “supervisor reports analysis” means (and in Fig 1). Please explain and also consider breaking this paragraph into more sentences for clarity.
- Table 7: please describe what 0-1 refers to, here and in the text. If this refers to dichotomous response options, consider presenting the proportion of respondents that gave a positive response as a percentage, along with the 95% CI, rather than presenting the mean and standard deviation.

DISCUSSION

- At times in this section, data is shared again, but summarizing without repeating the numeric results would make the section clearer
- There is a lot of information shared in the Discussion section about the history and development of UMHT, which may be better placed in the introduction section

*A*cceptability

- P. 13, 2 ^nd^ paragraph in section: please explain what “being in average high” means
- P. 13, 3 ^rd^ paragraph in section: this is a long sentence and difficult to understand; please rephrase and consider breaking into multiple sentences
- P. 13, 4 ^th^ paragraph: the first sentence (“Reports of perceived…”) is difficult to follow
- P. 13, last 3 paragraphs in section:  It is unclear when all of these actions were implemented – were they in response to feedback or present from the start?   
- P. 13, last paragraphs in section: Please provide a source/citation for the statement, “ *To pass through accreditation, a trainer should score no less than 3.5 subject knowledge”*

Extendability

- P.14, 1 ^st^ paragraph in section: UMHT steps are described here, but would be more appropriate in the introduction section 

Limitations and future research

- Please consider including additional limitations, such as 1) potential threats to internal validity from self-report using single-item measures, 2) limits of external validity based on the participant characteristics, and 3) lack of reliability and validity information for the novel measures used, which could present some concerns about measurement error.
- P. 15, 1 ^st^ paragraph: recommend rephrasing the first sentence for clarity, for example:
  “The main limitation of the performed feasibility research is the absence of effectiveness data of the impact of UMHT on recipients’ mental health and related outcomes *.” *

Is the study design appropriate and does the work have academic merit?

Yes

Is the work clearly and accurately presented and does it cite the current literature?

Partly

If applicable, is the statistical analysis and its interpretation appropriate?

Partly

Are all the source data underlying the results available to ensure full reproducibility?

Yes

Are the conclusions drawn adequately supported by the results?

Partly

Are sufficient details of methods and analysis provided to allow replication by others?

Partly

Reviewer Expertise:

clinical health psychology; task-shifting/task-sharing; implementation science

We confirm that we have read this submission and believe that we have an appropriate level of expertise to confirm that it is of an acceptable scientific standard, however we have significant reservations, as outlined above.

# Response

**Published:** 2025-01-08

**Authors:** Viktoriia Gorbunova

**Affiliations:** Department of Behavioural and Cognitive Sciences, University of Luxembourg, Esch-sur-Alzette, Luxembourg District, Luxembourg

We sincerely appreciate the feedback and concerns raised by the esteemed reviewers regarding our article. We accepted all proposed changes and implemented them in the new version of the article.

# Reviewer report: Reviewer response for version 1

**DOI:** 10.21956/openreseurope.18761.r42317

**Published:** 2024-09-20

**Authors:** Ling Wang

**Affiliations:** 1 King's College London, London, England, UK

Thanks for inviting this peer review. This is a timely work with rich data from the national level of implementation that could inform future frontline workforce to be prepared for mental health support. 

 Before meriting for indexing, please consider the following points to improve the readability of this work.

 Major Concerns

 1. Study Classification and Objectives

 The manuscript would benefit from a clear statement of the study type. Is this a process evaluation or a feasibility study? If it's the latter, please provide references for the feasibility indicators used and explain how they address each of the stated objectives. It will enhance the methodological rigour and align the study more closely with established research paradigms.

 2. Programme Description

 A significant omission is the lack of a comprehensive description of the universal mental health training programme for frontline professionals. Including a dedicated section detailing this national training project is crucial for understanding the context and potential applicability of the study findings.

 Minor Issues

 1. Knowledge Gap and Rationale: To strengthen the study's scientific value, please elaborate on:

- The rationale for implementing universal mental health training
- The necessity of conducting a feasibility study for this training programme
  This will help readers understand the study's significance within the broader context of mental health support and workforce development.

2. Background and Research Questions: I think we need to provide more background information on the design of this feasibility study, including the rationale for selecting the four domains of research questions. This will offer readers insight into the study's conceptual framework and methodological choices.

 3. Methods Section Structure: maybe could restructure the methods section for clarity:

1. Study design
2. Study setting or project overview
3. Data collection procedures
4. Analysis methods

This logical flow will enhance the readability and comprehension of the methodology.

 4. Results Presentation: In the results section, focus on reporting outcomes rather than reiterating measurement methods. For instance, instead of stating "We measured XXX for satisfaction," directly present the satisfaction results, which could probably reduce redundancy at some point.

 5. Discussion Structure: While the discussion of individual mental health and psychosocial support skills programmes is commendable, please consider a more holistic way synthesizing findings across programmes and drawing overarching conclusions.

 6. Also, if there are bit more comparisons with relevant literature, it will contextualize your findings within the broader field of mental health training and support.

 7. Conclusion and Background Integration: Consider moving some content from the third paragraph of the conclusion to the background section. This will ensure that the conclusion focuses on summarizing key findings and implications, while the background provides a comprehensive context for the study.

 The manuscript will likely achieve greater clarity, methodological rigour, and scholarly impact by addressing these points if these revisions can be done.

Is the study design appropriate and does the work have academic merit?

Partly

Is the work clearly and accurately presented and does it cite the current literature?

Yes

If applicable, is the statistical analysis and its interpretation appropriate?

Yes

Are all the source data underlying the results available to ensure full reproducibility?

Yes

Are the conclusions drawn adequately supported by the results?

Yes

Are sufficient details of methods and analysis provided to allow replication by others?

Yes

Reviewer Expertise:

mental health and psychosocial support during emergencies, implementation science

I confirm that I have read this submission and believe that I have an appropriate level of expertise to confirm that it is of an acceptable scientific standard, however I have significant reservations, as outlined above.

# Response

**Published:** 2024-10-17

**Authors:** Viktoriia Gorbunova

**Affiliations:** Department of Behavioural and Cognitive Sciences, University of Luxembourg, Esch-sur-Alzette, Luxembourg District, Luxembourg

We sincerely appreciate the feedback and concerns raised by the esteemed reviewer regarding our article. We have clarified them and pointed out the amendments that will be made.    

*1. Study Classification and Objectives*

*The manuscript would benefit from a clear statement of the study type. Is this a process evaluation or a feasibility study? If it's the latter, please provide references for the feasibility indicators used and explain how they address each of the stated objectives. It will enhance the methodological rigour and align the study more closely with established research paradigms.*   

 A feasibility study and a feasibility analysis (the term used in our article title and the text) are often used interchangeably, and refer to the same process. Both terms describe a systematic evaluation of a proposed project to determine its viability. Thank you for raising the issue; we will make the study type more visible in the Methods section in the next article version, highlighting it in the words “feasibility study”. As for the feasibility indicators, we use the term feasibility focus areas as proposed in Bowen DJ, Kreuter M, Spring B, et al.: How we design feasibility studies. Am J Prev Med. 2009; 36(5): 452–457. To avoid misreading and setting the frame of study reporting, we will add the source reference right after the first mention of feasibility focus areas in the relevant section, “Research methods and measurements”. The feasibility indicators (focus areas) themselves are available in Table 2. Namely, Demand (explored via usability statistics); Acceptability (explored via satisfaction surveying and accreditation structured assessment); Adaptability (explored via usability surveying, supervision reports evaluation, and case comparison); and Extendibility (explored via usability surveying, supervision reports evaluation, and case comparison).    

*2. Programme Description* 

*A significant omission is the lack of a comprehensive description of the universal mental health training programme for frontline professionals. Including a dedicated section detailing this national training project is crucial for understanding the context and potential applicability of the study findings.*   

 The extended description was provided in the previous article (https://open-research-europe.ec.europa.eu/articles/4-19), referred to in the Introduction section of the current text. However, we agree that this reference and additional programme description must be reiterated for the reader’s convenience in the Method section of this article. It will be addressed in the next iteration of the article.   

*3. To strengthen the study's scientific value, please elaborate on the rationale for implementing universal mental health training.*   

 The rationale for implementation is presented in the previous article (https://open-research-europe.ec.europa.eu/articles/4-19), referred to in the Introduction section.   

*4. To strengthen the study's scientific value, please elaborate on the necessity of conducting a feasibility study for this training programme. This will help readers understand the study's significance within the broader context of mental health support and workforce development. *   The question is addressed in the Introduction section: "The feasibility of such public health programmes as the UMHT is one of the primary requirements for their implementation (Bowen et al., 2009). In every case, a feasibility study answers whether an intervention is appropriate for further development, testing and implementation and identifies if anything needs modification and adjustments. According to the Framework for the Development and Evaluation of Complex Interventions issued by the National Institute for Health and Care Research (NIHR), research-driving policymaking implies feasibility and pilot trials as steps taken immediately after intervention development prior to effectiveness evaluation (Skivington et al., 2021)."   

*5. Background and Research Questions: I think we need to provide more background information on the design of this feasibility study, including the rationale for selecting the four domains of research questions. This will offer readers insight into the study's conceptual framework and methodological choices.*   

 We agree. It will be addressed in the next iteration of the article.   

*6. Methods Section Structure: maybe could restructure the methods section for clarity: Study design, Study setting or project overview, Data collection procedures, Analysis methods. This logical flow will enhance the readability and comprehension of the methodology.*   

 In the next iteration of the article, we will restructure the Methods Section according to your suggestion, keeping in mind the chosen frame of study reporting.   

*7. Results Presentation: In the results section, focus on reporting outcomes rather than reiterating measurement methods. For instance, instead of stating "We measured XXX for satisfaction," directly present the satisfaction results, which could probably reduce redundancy at some point.*   

 We agree the issue will be addressed in the next iteration of the article.   

*8. Discussion Structure: While the discussion of individual mental health and psychosocial support skills programmes is commendable, please consider synthesizing findings across programmes in a more holistic way and drawing overarching conclusions.*   

 The Discussion section is built around all four feasibility indicators (focus areas) and goes beyond the MHPSS programmes (in the Demand subsection). Further section conceptualisation and ideas are presented in the subsections on Demand, Acceptability, Adaptability, and Extendibility focus areas.    

*9. Also, if there are bit more comparisons with relevant literature, it will contextualize your findings within the broader field of mental health training and support.*   

 It will be considered in the next iteration of the article.   

*10. Conclusion and Background Integration: Consider moving some content from the third paragraph of the conclusion to the background section. This will ensure that the conclusion focuses on summarizing key findings and implications, while the background provides a comprehensive context for the study.*   

 The issue will be addressed in the next iteration of the article.

# Response

**Published:** 2025-01-08

**Authors:** Viktoriia Gorbunova

**Affiliations:** Department of Behavioural and Cognitive Sciences, University of Luxembourg, Esch-sur-Alzette, Luxembourg District, Luxembourg

We sincerely appreciate the feedback and concerns raised by the esteemed reviewer regarding our article. Further, we clarified them and pointed out the amendments that will be made.   1. Study Classification and Objectives The manuscript would benefit from a clear statement of the study type. Is this a process evaluation or a feasibility study? If it's the latter, please provide references for the feasibility indicators used and explain how they address each of the stated objectives. It will enhance the methodological rigour and align the study more closely with established research paradigms.  

- Regarding the Study Classification and Objectives, the study type is a feasibility study. A feasibility study and a feasibility analysis (the term used in our article title and in the text) are often used interchangeably, and refer to the same process. Both terms describe a systematic evaluation of a proposed project to determine its viability.
- As for the feasibility indicators we use term feasibility focus areas as proposed in Bowen DJ, Kreuter M, Spring B, et al.: How we design feasibility studies. Am J Prev Med. 2009; 36(5): 452–457. To avoid misreading and set the frame of study reporting we will add the source reference right after first mentioning of feasibility focus areas in the relevant section “Research methods and measurements” and will mention the focus areas in the abstract. The feasibility indicators (focus areas) themselves are available in Table 2. Namely, Demand, explored via the usability statistic; Acceptability, explored via the satisfaction surveying and accreditation structured assessment, adaptability, explored via usability surveying, supervision reports evaluation, case comparison and extendability explored via usability surveying, supervision reports evaluation, and case comparison.

  2. Programme Description A significant omission is the lack of a comprehensive description of the universal mental health training programme for frontline professionals. Including a dedicated section detailing this national training project is crucial for understanding the context and potential applicability of the study findings.  

- The extended description was provided in the previous article (https://open-research-europe.ec.europa.eu/articles/4-19), which is referred to in the Introduction section of the current text. We will add a description of the programme’s essence and highlight the reference to the previous article as a must-see in the next iteration of the article.

  3. To strengthen the study's scientific value, please elaborate on the rationale for implementing universal mental health training  

- The rationale for implementing is presented in the previous article (https://open-research-europe.ec.europa.eu/articles/4-19), which is referred to in the Introduction section.  We will highlight the reference to the previous article as a must-see in the next iteration of the article.

  4. To strengthen the study's scientific value, please elaborate on the necessity of conducting a feasibility study for this training programme. This will help readers understand the study's significance within the broader context of mental health support and workforce development.  

- The question is addressed in the Introduction section: The feasibility of such public health programmes as the UMHT is one of the primary requirements for their implementation (Bowen et al., 2009). In every case, a feasibility study answers whether an intervention is appropriate for further development, testing and implementation and identifies if anything needs modification and adjustments. According to the Framework for the Development and Evaluation of Complex Interventions issued by the National Institute for Health and Care Research (NIHR), research-driving policymaking implies feasibility and pilot trials as steps taken immediately after intervention development prior to effectiveness evaluation (Skivington et al., 2021).

  5. Background and Research Questions: I think we need to provide more background information on the design of this feasibility study, including the rationale for selecting the four domains of research questions. This will offer readers insight into the study's conceptual framework and methodological choices.  

- We agree. It will be addressed in the next iteration of the article.

  6. Methods Section Structure: maybe could restructure the methods section for clarity: Study design, Study setting or project overview, Data collection procedures, Analysis methods. This logical flow will enhance the readability and comprehension of the methodology.  

- We will restructure the Methods Section according to your suggestion in the next iteration of the article.

  7. Results Presentation: In the results section, focus on reporting outcomes rather than reiterating measurement methods. For instance, instead of stating "We measured XXX for satisfaction," directly present the satisfaction results, which could probably reduce redundancy at some point.  

- Thank you for the suggestion. However, it might disrupt the logic of the presentation of the results.

  8. Discussion Structure: While the discussion of individual mental health and psychosocial support skills programmes is commendable, please consider synthesizing findings across programmes in a more holistic way and drawing overarching conclusions.  

- The Discussion section is built around all four feasibility indicators (focus areas) and goes beyond the MHPSS programmes (in the Demand subsection). Further section conceptualisation and ideas are presented in the subsections on Acceptability, Adaptability, and Extendability focus areas.

  9. Also, if there are bit more comparisons with relevant literature, it will contextualize your findings within the broader field of mental health training and support.  

- We agree that the wide contextualisation of MH programmes is important and will consider writing a separate article dedicated to this aim. The aim of the current one was to analyse the viability of the specific public mental health programme for the particular country and its context. That is why for the contextual analysis were chosen feasibility studies of widespread public mental health programmes such as Psychological First Aid / PFA,  Mental Health First Aid / MHFA and Mental Health Gap Action Programme / mhGAP.

  10. Conclusion and Background Integration: Consider moving some content from the third paragraph of the conclusion to the background section. This will ensure that the conclusion focuses on summarizing key findings and implications, while the background provides a comprehensive context for the study.  

- The issue will be addressed in the next iteration of the article.
